# Transcriptomes shed light on transgenerational and developmental effects of ocean warming on embryos of the sea urchin *Strongylocentrotus intermedius*

**DOI:** 10.1038/s41598-020-64872-x

**Published:** 2020-05-13

**Authors:** Dongtao Shi, Chong Zhao, Yang Chen, Jingyun Ding, Lisheng Zhang, Yaqing Chang

**Affiliations:** Key Laboratory of Mariculture & Stock Enhancement in North China’s Sea, Ministry of Agriculture and Rural Affairs, Dalian Ocean University, Dalian, 116023 China

**Keywords:** Ecological genetics, Marine biology

## Abstract

Ocean warming increasingly endangers the fitness of marine invertebrates. Transgenerational effects (TE) potentially mitigate the impacts of environmental stress on the embryos of marine invertebrates. The molecular mechanisms, however, remain largely unknown. Using high-throughput RNA sequencing technology, we investigated the gene expression patterns of embryos (the gastrula stage) of the sea urchin *Strongylocentrotus intermedius* at different developmental temperatures, whose parents were exposed to long-term (15 months) elevated temperature (A) or not (B). The temperatures at which adults were held for ~4 weeks prior to the start of the experiment (21 °C for A and 18 °C for B) were also used for the development of offspring (high: 21 °C and ambient (laboratory): 18 °C) resulting in four experimental groups (HA and HB at 21 °C, and LA and LB at 18 °C). The embryos were sampled ~24 h after fertilization. All samples were in the gastrula stage. Twelve mRNA libraries (groups HA, HB, LA, LB, 3 replicates for each group) were established for the following sequencing. Embryos whose parents were exposed to elevated temperatures or not showed 1891 significantly different DEGs (differentially expressed genes) at the ambient developmental temperature (LB vs LA, LB as control) and 2203 significantly different DEGs at the high developmental temperature (HB vs HA, HB as control), respectively. This result indicates complex molecular mechanisms of transgenerational effects of ocean warming, in which a large number of genes are involved. With the TE, we found 904 shared DEGs in both LB vs LA (LB as control) and HB vs HA (HB as control) changed in the same direction of expression (i.e., up- or down-regulated), indicating that parental exposed temperatures affect the expression of these genes in the same manner regardless of the development temperature. With developmental exposure, we found 198 shared DEGs in both HB vs LB (HB as control) and HA vs LA (HA as control) changed in the same direction of expression. Of the 198 DEGs, more genes were up-regulated at high developmental temperature. Interestingly, embryos whose parents were exposed to high temperature showed fewer differently expressed DEGs between high and low developmental temperatures than the individuals whose parents were exposed to ambient temperature. The results indicate that gene expressions are probably depressed by the transgenerational effect of ocean warming. The roles of *hsp70* and *hnf6* in thermal acclimation are highlighted for future studies. The present study provides new insights into the molecular mechanisms of the transgenerational and developmental effects of ocean warming on the embryos of sea urchins.

## Introduction

Ocean warming is an increasing threat to the fitness of marine invertebrates because the water temperature is predicted to rise 2–4 °C over the next century by the Intergovernmental Panel on Climate Change (IPCC)^1^. Marine invertebrates either acclimatize and/or adapt to natural changes or migrate to avoid rising temperatures^2–4^. Those with weak capacity for mobility in shallow water are more susceptible and consequently necessarily require quick acclimation and/or adaptation to ocean warming^5,6^. Transgenerational effect (TE) is an across generational carryover effect, in which the phenotype of offspring changes in response to the exposure of one or both parents to various environments^7^. Transgenerational effect plays a potential role in marine organisms responding to temperature changes^7–10^. For example, the thermal limit of the sea urchin *Heliocidaris erythrogramma* was significantly affected by the thermal experience of their parents^11^. However, the molecular mechanisms of the TE of ocean warming remain largely unknown in marine invertebrates. Transcriptomics is an effective method to reveal the molecular mechanisms of TE in changing environments and thus provides valuable information about the molecular mechanisms of TE in marine invertebrates^5,12,13^.

**Figure 1:**
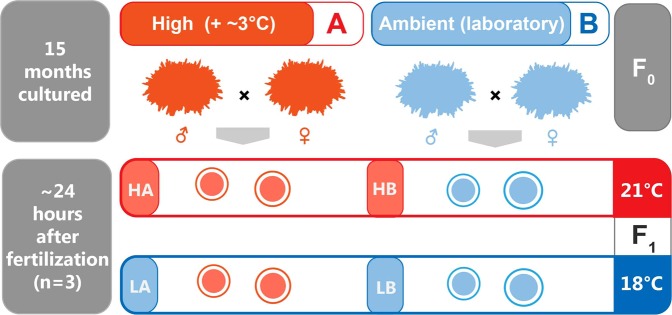
Experimental design for the transgenerational and developmental temperature effects on gene expressions of *Strongylocentrotus intermedius*. (**A,B**) refer to parents exposed to the elevated (~+3 °C) and ambient temperatures. HA and LA represent the offspring of group A at high and ambient developmental temperatures, respectively. HB and LB were designated accordingly.

Sea urchins living in shallow water are susceptible to environmental influences and are good experimental animals for TE. The development of their embryo is significantly affected by high temperatures, greatly affecting their continuation^11,14^. Our previous study found negative transgenerational effects in hatchability and most traits of larval size of the sea urchin *Strongylocentrotus intermedius* whose parents were exposed to a + ~3 °C temperature for ~15 months^15^. This clearly indicates the TE of ocean warming on embryo development and larval size of *S. intermedius*. To reveal the molecular basis of this phenomenon, we accordingly used our previous experimental design for further investigation, in which parental *S. intermedius* were exposed to long-term elevated temperature (+~3 °C, A) or not (B) for ~15 months before the TE experiments. Fertilized eggs were held at two development temperatures (high at 21°C and ambient (laboratory) at 18°C) (Fig. 1). In addition, the abundant genetic information of embryo development can greatly support analyses after the sequencing in the present investigation, because sea urchin is a well-known research model of developmental biology. For example, hepatocyte nuclear factor (*hnf6*)^16^ and heat shock protein (*hsp70*)^17^ have been well documented in sea urchin development^15,18,19^. The main purposes of the present study are to investigate 1) how TE of high temperature affects gene expressions of embryos of *S. intermedius* (transgenerational effect) and 2) how the developmental temperature influences gene expressions of embryos of *S. intermedius* (developmental temperature effect).

## Results

### Transcriptome sequencing and unigenes functional annotation

After trimming the sequence reads, the number of reads per sample was from 41,629,836 to 42,511,950. Clean reads ratio accounted for 94.30%. After screening, 505,655,384 clear reads were obtained, of which Q30 and GC accounted for 88.27% and 39.42%, respectively. The total length was 95,978,844 bp with an average length of 1332.04 bp and an N50 of 2121 bp from the *de novo* assembly (Table S1). The assembled genes were deemed 94.4% completed by BUSCO, including 80.2% as single genes and 14.2% as duplicated genes. These results showed that the sequencing quality was sufficient for further bioinformatics analyses. The principal component analysis (PCA) of the expression of unigenes was shown in Fig. S1 for the better understanding of the parental effects (N = 3).

### Gene annotation and classification

Unigenes of all transcripts were annotated through the databases of NR, SWISSPROT, and KOG, with a cut of e < 1e^−5^. The NR database annotated 30.36% (21,873) of the genes. The numbers of annotations were 14,547 (20.19%) and 12,050 (16.72%) using the SWISSPROT and KOG databases, respectively (Table S2). The top categories of KOG were general function prediction only, signal transduction mechanisms and posttranslational modification, protein turnover, chaperones (Fig. S2).

Most of the unigenes (13,148; 18.25%) obtained were distributed in 62 GO terms into three categories of biological process (23), cellular component (19), and molecular function (20), basing on the results of blast NR (Fig. S3). A total number of 4577 (6.35%) unigenes were assigned to 359 pathways in the KEGG pathway analysis.

### DEGs among experimental groups

**Figure 2:**
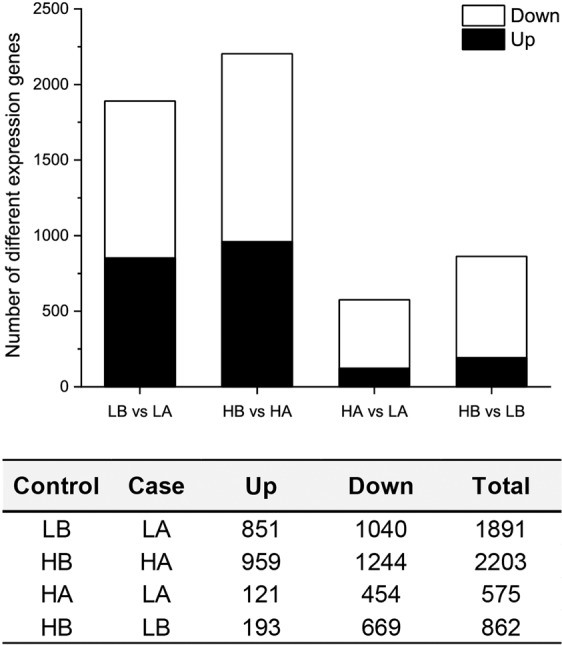
Number of differentially expressed genes among experimental groups of *Strongylocentrotus intermedius*. (**A,B**) refer to parents exposed to the elevated and ambient temperatures. HA and LA represent the offspring of group A at high and ambient developmental temperatures, respectively. HB and LB were designated accordingly.

**Figure 3:**
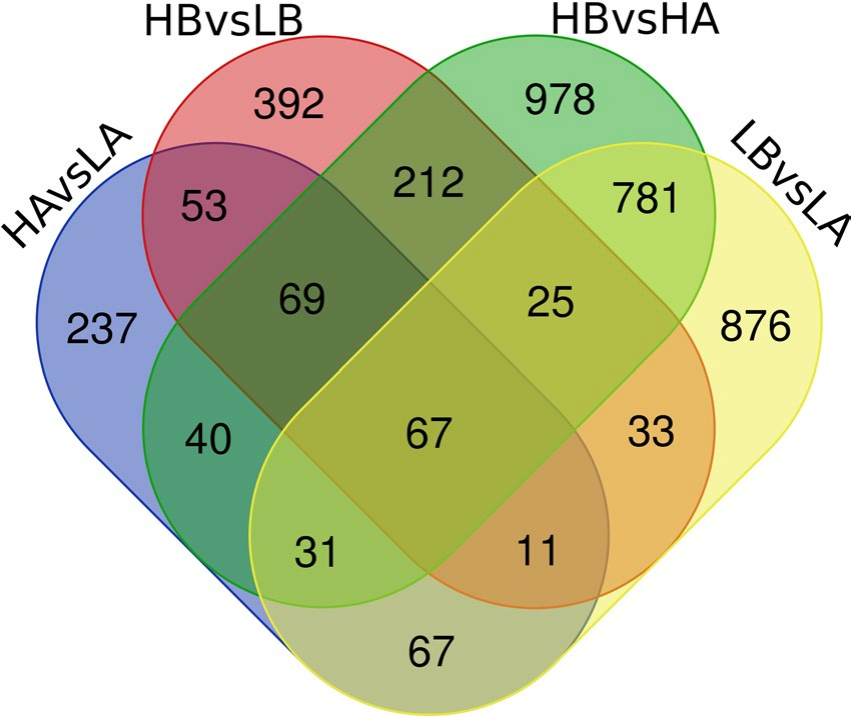
Difference of gene expression among experimental treatments of *Strongylocentrotus intermedius*. A and B refer to parents exposed to the elevated and ambient temperatures. HA and LA represent the offspring of group A at high and ambient developmental temperatures, respectively. HB and LB were designated accordingly.

With TE, 1891 DEGs showed a significant difference in gene expression between LB vs LA, in which 851 were up-regulated and 1040 down-regulated. Comparatively, we found 2203 differently expressed DEGs between HB vs HA, in which 959 up-regulated and 1244 down-regulated (Fig. 2). There were 904 DEGs (377 up-regulated and 527 down-regulated) were intersected between LB vs LA and HB vs HA (Fig. 3). Interestingly, we found *hnf6* was up-regulatory expressed in LB vs LA and HB vs HA, regardless of the developmental temperature. However, *hnf6* showed no significantly different expression in either HA vs LA or HB vs LB.

With developmental exposure, 575 genes of embryos, whose parents were exposed to an elevated temperature for ~15 months, differentially expressed at different developmental temperatures (HA vs LA), in which 121 genes were up-regulated and 454 down-regulated (Fig. 2). There were 862 differently expressed DEGs (193 up-regulated and 669 down-regulated) between HB and LB. Further, we found 200 DEGs (9 up-regulated, 189 down-regulated, and 2 differently regulated) significantly expressed at the intersection of HA vs LA and HB vs LB, regardless of the parental exposed temperatures (Fig. 3). *Hsp70* showed significantly higher expression in HB than in LB, while there was no difference of gene expression between HA and LA.

GO classification of DEGs among different groups was shown in Table S3. The TEs of ocean warming were explored by HB vs HA (595 and 1462 terms enriched up- and down-regulated genes) and LB vs LA (472 and 1186 terms enriched up- and down-regulated genes) in GO. We found that most up-regulated DEGs in both HB vs HA and LB vs LA were annotated in the nucleus, RNA-directed DNA polymerase activity, and DNA binding. Most down-regulated DEGs (both HB vs HA and LB vs LA) were classified into an integral component of membrane, plasma membrane, and calcium ion binding terms, regardless of the developmental temperature. The 904 shared DEGs were mainly associated with sodium channel activity, nucleosome, and carboxypeptidase activity in GO terms.

To explore the embryo developmental temperature effects, we compared HA vs LA (92 and 754 terms enriched up- and down-regulated genes) and HB vs LB (303 and 1123 terms enriched up- and down-regulated genes), respectively. More DEGs were enriched in HB vs LB than in HA vs LA. In both comparisons, more down-regulated DEGs were enriched in GO than up-regulated DEGs. Most up-regulated DEGs of both HB vs LB and HA vs LA were enriched in the nucleus, nucleolus, and poly(A) RNA binding terms, while most down-regulated DEGs were classified into the integral component of membrane, plasma membrane and calcium ion binding entries. Of the 198 shared genes (with same regulated direction), most down-regulated genes were classified into neurotransmitter catabolic process, carboxypeptidase activity, metallopeptidase activity, and calcium ion binding, while most up-regulated genes were classified into cell junction.

## Discussion

Long-term thermal experience of parents has significant impacts on the embryo development of sea urchins^7,15,18^. Our previous study, for example, showed that the embryo development was significantly influenced at ~27 hours after fertilization in *S. intermedius*, whose parents were exposed to long-term elevated temperature^15^. The molecular basis, however, remains mostly unknown. In the present study, embryos whose parents were exposed to elevated temperatures or not showed 1891 significantly different DEGs at the ambient temperature (LB vs LA) and 2203 significantly different DEGs at the high temperature (HB vs HA), respectively. This result indicates complex molecular mechanisms of transgenerational effects of ocean warming on embryo development, in which a large number of genes are involved. Together with the previous information^20^, the present transcriptomic investigation on long-term parental exposure to high temperature enriches our understanding of the molecular basis of transgenerational effects of ocean warming on sea urchins.

Interestingly, we found all the 904 shared DEGs in both LB vs LA and HB vs HA were changed in the same expression direction (i.e., up- or down-regulated), indicating that the manner of gene expression was related to the parental exposed temperature, regardless of the developmental temperature. Consistent with our finding, Runcie *et al*. (2012) indicates that parents have more influences on offspring than the developmental environments^19^. Further, we found 22 DEGs (15 down-regulated and 7 up-regulated) related to the development and growth of embryos. These genes probably provide the molecular basis for the consequent transgenerational influences on the larval development of *S. intermedius*, regardless of developmental temperatures^15^. Down-regulation of more genes may be an important reason for interfering with larval development^15^. Further, the 904 DEGs were mainly associated with sodium channel activity, nucleosome, and carboxypeptidase activity in GO terms. Nuclear activity is essential for the gastrulation of sea urchins^12,21^. In the present study, the up-regulated DEGs (HB vs HA and LB vs LA) were highly related to nuclear activity, suggesting the importance in TE. This result, however, is not consistent with a *p*CO_2_ stress study by Wong *et al*. (2018), in which nuclear activity was not affected by maternal effects^12^. This disagreement is probably due to the differences in adult treatments using multiple stressors (water temperature and *p*CO_2_) versus a single stressor (water temperature only), or species differences. Sea urchin larvae transport ions through their cell membranes to modulate their homeostasis in response to environmental changes (such as *p*CO_2_)^22^. Unsurprisingly, ion transportation in the TE was also found in the study of Wong *et al*.^12^. In the present study, most down-regulated DEGs were involved in the calcium ion binding, and most up-regulated DEGs ware in zinc ion binding in the embryos whose parents experienced the high temperature, regardless of the developmental temperature. This indicates that the ion homeostasis was well regulated in the embryos whose parents were exposed to long-term elevated temperatures.

A large number of genes (such as *hnf6*) expressed zygotically in the embryos of the maternal transcriptome^16^. Thus, the environmental stress that their parents are exposed to probably has a larger impact on these genes. *Hnf6* is important in the early development of sea urchins^16^. Like other transcription factors, *hnf6* as an essential participant in the multiple regulatory networks involved in the embryo development of sea urchins^16,23^. *Hnf6* expression is essential for maintaining the oral ectoderm specification status in the oral ectoderm gene regulatory network of embryos of sea urchins^16,19^. In the present study, *hnf6* expression showed a significant difference in both HB vs HA and LB vs LA, but no significant difference in neither HA vs LA nor HB vs LB. The result indicates that *hnf6* expression is regulated by the thermal experience of their parents, rather than by the developmental temperature. Interestingly, *hnf6* expression of the sea urchin *Strongylocentrotus purpuratus* was significantly lower at 18 °C than that at 15 °C^19^. In our previous study, the development rate of larvae in group A was lower than that in group B in the ~27 hours developmental condition, regardless of temperature^15^. This phenomenon can be explained by the significant difference in *hnf6* expression. The TE regulated *hnf6* expression, therefore, probably performs further influences through the complex gene regulatory network.

Water temperature is an essential factor affecting the development of sea urchin embryos, including growth^15,18^ and gene expression^19^. Elevated temperature significantly decreased the cleavage (+4–6 °C) and normal gastrulation (+6 °C) of the sea urchin *Heliocidaris erythrogramma*^24^. Further, extensive changes in gene expression of sea urchin embryos, including protein folding, RNA processing, and development, were triggered by +6 °C (from 12 °C to 18 °C)^24^. The present study found that the gene expression consistently increased when embryos were developed at 21 °C compared with those at 18 °C, regardless of whether their parents were exposed to long-term elevated temperature or not. This finding is consistent with a previous finding that a high temperature increased the gene expression activity of the sea urchin *S. purpuratus*^19^. Our results identified 198 shared DEGs with the same trend of gene expression in both HA vs LA and HB vs LB, with 12 of them were related to the development of sea urchins. This suggests that these genes probably play an essential regulatory role in the embryo development of sea urchins at high temperatures. Of the 198 DEGs, many genes were up-regulated at high developmental temperature. They were associated with neurotransmitter catabolic process, carboxypeptidase activity, metallopeptidase activity, and calcium ion binding in GO terms. A reasonable explanation for this result is that the up-regulated expressions support the high temperature promoted growth process of sea urchins^14^. Another possible explanation is that these up-regulated genes are associated with a heat stress response at the elevated developmental temperature. Notably, we probably underestimated the effects of developmental heterogeneity induced by developmental temperature on the present results, although all embryos were approximately in the stage of the gastrula.

High developmental temperature increased gene expression of calcium ion binding (involved in multiple biological effects and cell signaling processes)^25–27^ and serine-type endopeptidase activity (involved in the early development)^28^. The increased gene expression could promote the development of sea urchins. However, all offspring died nine days after fertilization at 21 °C, regardless of their parental exposure temperatures^15^. Interestingly, we found that embryos whose parents were exposed to high temperature showed fewer differently expressed genes between high and low developmental temperatures than the individuals whose parents were exposed to ambient temperature. The gene expression depressed by the TE of ocean warming can be explained by the epigenetic regulation of gene expression^7,13,29^. Hsp 70, which is an important component in cellular protein folding^30,31^, is well known for an increased expression at high temperature^19^. The expression of *hsp70* in the gastrula stage was significantly higher at elevated temperature (+4 °C) than that at ambient temperature^32^. These studies are consistent with our finding that *hsp70* expression significantly increased at high temperature when their parents were exposed to ambient temperature. However, *hsp70* did not increase when their parents were exposed to elevated temperature. This result indicates a negative TE of ocean warming on *hsp70* expression of sea urchin embryos, which could subsequently affect the survival of sea urchin larvae under ocean warming^13^.

In conclusion, the present study indicates complex molecular mechanisms of transgenerational effects of ocean warming on embryos (the gastrula stage) of the sea urchin *S. intermedius*, in which a large number of genes are involved. The parental exposed temperature affected the expression of these genes in the same trend regardless of the developmental temperature. Interestingly, parental exposed temperature showed greater influence on embryo gene expression than the developmental temperature. Our study further indicates that gene expressions are probably depressed by the TE of ocean warming. The roles of *hsp70* and *hnf6* in thermal acclimation are highlighted for future studies. Notably, the effects of families and genotypes are probably involved in the gene expressions. The present study provides new insights into the molecular mechanisms of the transgenerational and developmental effects of ocean warming on the embryos of sea urchins.

## Materials and methods

### Sea urchins and experimental design

The source of the parental sea urchins, temperature treatments, and embryo breeding were fully described in our previous studies^15,33^ and briefly summarized as follows:

Parental *S. intermedius* were exposed to long-term elevated temperature (+~3 °C, A) or not (B) for ~15 months (from July 8, 2015 to October 19, 2016) before the TE experiments. Parental *S. intermedius* were acclimated for 4 weeks at ~18 °C for group B and ~21 °C for group A, respectively. Breeding and subsequent embryo incubation were carried out in the natural spawning season of *S. intermedius* (15.2–18.5 °C is the suitable temperature range for the natural reproduction)^34,35^ and at suitable spawning temperature (~18 °C for group B and thus ~21 °C for group A). Two adults (a sire and a dam) bred one replicate (e.g., in group A), which was subsequently hatched at two temperatures (e.g., HA and LA). Two different adults (a sire and a dam) established another replicate, and so on. Three families were established for either group A or group B using different parental sea urchins according to the experimental design (N = 3). Twelve tanks were consequently used for groups A and B at two development temperatures (HA, LA, HB and LB, N = 3). HA and LA represent the offspring of group A at high and ambient developmental temperatures, respectively. HB and LB were designated accordingly (N = 3, Fig. 3).

### Sample collection and RNA extraction

About 12 μg larvae were sampled in each of the 12 tanks for the following RNA-seq ~24 hours after fertilization. We collected the larvae that had already floated when we collected the samples, which can exclude some lower developmental stages. To ensure the developmental consistency of the sampled embryos, we measured the development stages using a microscope (DS-Ri1, Canon, Japan). All embryos from the 12 tanks were in the gastrula stage at ~27 hours after fertilization (Fig. S4). Samples were centrifuged at 2000 × rpm for 2 minutes (4 °C) to harvest embryos that were subsequently placed into liquid nitrogen and stored at −80 °C. The total RNA of the samples was extracted using Ambion-1561 according to the instructions of the manufacturer. The quality and quantity of RNA evaluated by 1% agarose gel electrophoresis and UV spectrophotometry, respectively.

### Libraries construction and high-throughput sequencing

Twelve libraries were constructed using TruSeq Stranded mRNA LT Sample Prep Kit (Illumina, San Diego, CA, USA) according to the manufacturer’s instructions. Four μg RNA per sample was used as the input material for RNA preparation. mRNA was purified by adding 50 µL RNA purification beads. The first strand cDNA was synthesized using the First Strand Synthesis Act D Mix and Super Script II Reverse. The second strand cDNA was synthesized by Second Strand Marking Master Mix. The mixture was incubated after adding the adenylate 3′ and then 2.5 μL diluted A-Tailing Control (1 μL A-Tailing Control + 99 μL Resuspension Buffer). Thirty µl supernatant was collected after purification, enrichment, and repurification. One μL sample was loaded on an Agilent 2100 Bioanalyzer to check the size and purity of the libraries. The twelve libraries were sequenced using the Illumina sequencing platform (HiSeq TM 2500). Paired-end reads of 150 bp/125 bp were generated. Following sequencing, quality control of the raw data was performed using the software FastQC v0.10.1. GC content and sequence replication level of Q30 were subsequently calculated. The raw data were subjected to the quality preprocessing by filtering low quality reads (quality threshold 20, length threshold 35 bp), to remove low-quality bases from the 3′ end (quality threshold 20), and to excise read containing N partial sequences (length threshold 35 bp). The number of reads in the whole quality control process was statistically summarized.

### Transcriptome assembly and annotation

Transcriptomes were assembled using the paired-end method of Trinity (trinityrnaseq_r20131110)^36^. According to the sequence similarity and length, the longest one was selected as unigenes. TGICL software clustering was subsequently used to remove redundancy and to obtain a final set of unigenes, which was as a reference sequence for subsequent analysis^37^. BUSCO v.3.0.0 (e-value = 1e^−3^) was used to assess the completeness of the gene assembly^38^. The unigene sequences were compared with public databases by blastx, including NCBI non-redundant protein sequences (NR), a manually annotated and reviewed protein sequence database (SWISSPROT), and Clusters of Orthologous Groups of proteins (KOG). The annotations of e < 1e^−5^ were taken to obtain the proteins with the highest sequence similarity to given unigenes. We performed GO^39^ (Blast2GO v2.5, default parameters) functional significance and KEGG^40^ (KAAS, KEGG Automatic Annotation Server, default parameters) for the pathway enrichment analyses of the differentially expressed genes (DEGs).

The significantly different DEGs were calculated using the negative binomial distribution test in the DESeq software (padj < 0.05 and |foldchange | > 2) (http://bioconductor.org/packages/release/bioc/html/DESeq.html)^41^. The negative binomial distribution (NB) and base mean values were used to test the difference in the number of reads and gene expression, respectively.

## Supplementary information

Supplementary Material.

## Data Availability

All sequence data were submitted to the NCBI Short-Read Archive (SRA) with the accession number PRJNA558427.
