# Supplementary material for: Transcriptomes shed light on transgenerational and developmental effects of ocean warming on embryos of the sea urchin Strongylocentrotus intermedius
Source: Sci Rep. 2020 May 13;10:7931. doi: 10.1038/s41598-020-64872-x (PMC7221070; doi:10.1038/s41598-020-64872-x)
Supplement: Supplementary file 1 — Supplementary Material. [file 41598_2020_64872_MOESM1_ESM.docx]

**Transcriptomes shed light on transgenerational and developmental effects of ocean warming on embryos of the sea urchin *Strongylocentrotus intermedius***

**Dongtao Shi, Chong Zhao^*^, Yang Chen, Jingyun Ding, Lisheng Zhang, Yaqing Chang^*^**

Key Laboratory of Mariculture & Stock Enhancement in North China’s Sea, Ministry of Agriculture and Rural Affairs, Dalian Ocean University, Dalian, 116023, China

^____________________________________________________________________________________________________^

**^*^ Correspondence:** Chong Zhao, Email: chongzhao@dlou.edu.cn; Yaqing Chang, Email: changlab@hotmail.com

**Supplementary Figure Legends**

**Figure S1** The principal component analysis (PCA) of the expression of unigenes. (N = 3)

**Figure S2** Clusters of orthologous groups for eukaryotic complete genomes (KOG) function classification of unigenes from the *Strongylocentrotus intermedius* transcriptomes. A total of 12,050 unigenes were assigned to 25 subclasses.

**Figure S3** Gene ontology (GO) classifications of annotated unigenes from the *Strongylocentrotus intermedius* transcriptomes.

**Figure S4** Developmental stages of embryos of *Strongylocentrotus intermedius* ~27 hours after the fertilization.

**Figure S1
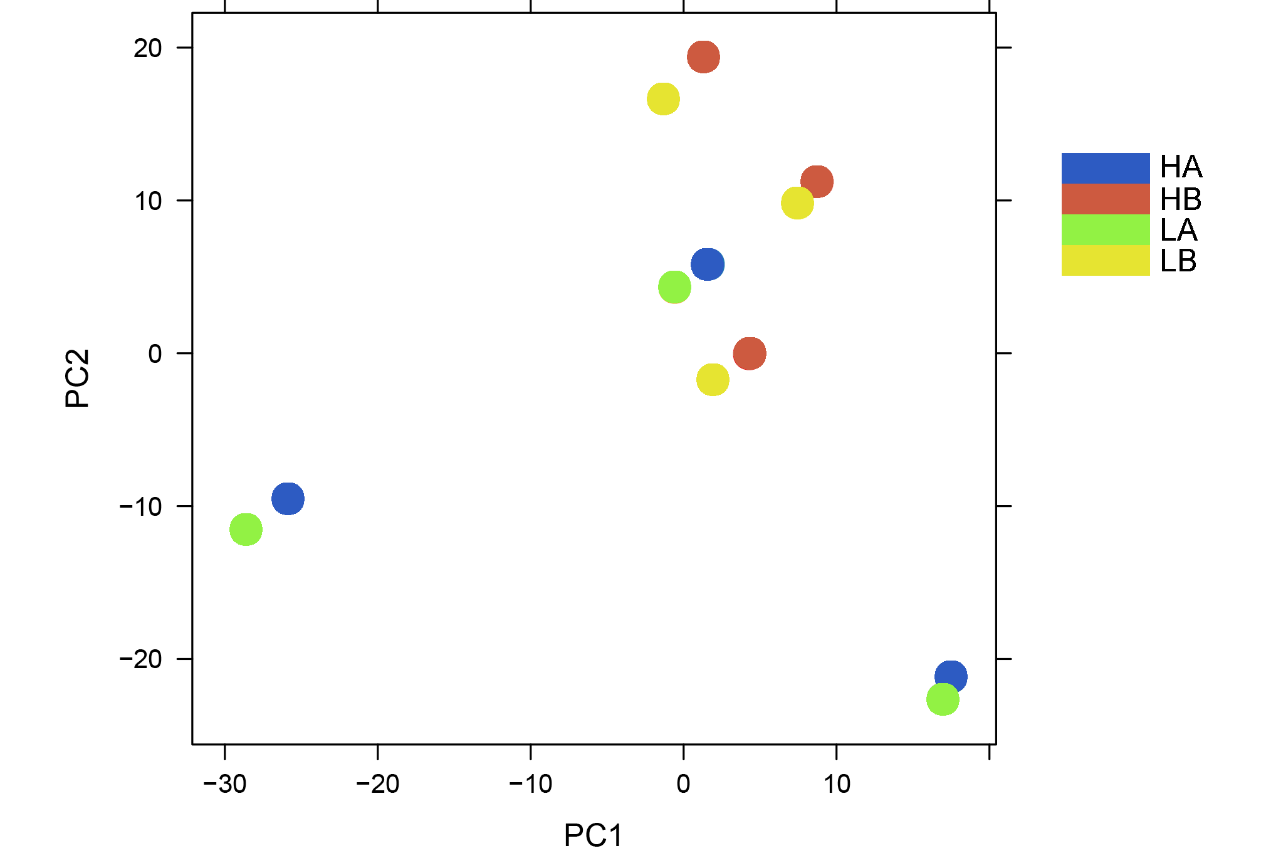
**

**Figure S2**

**
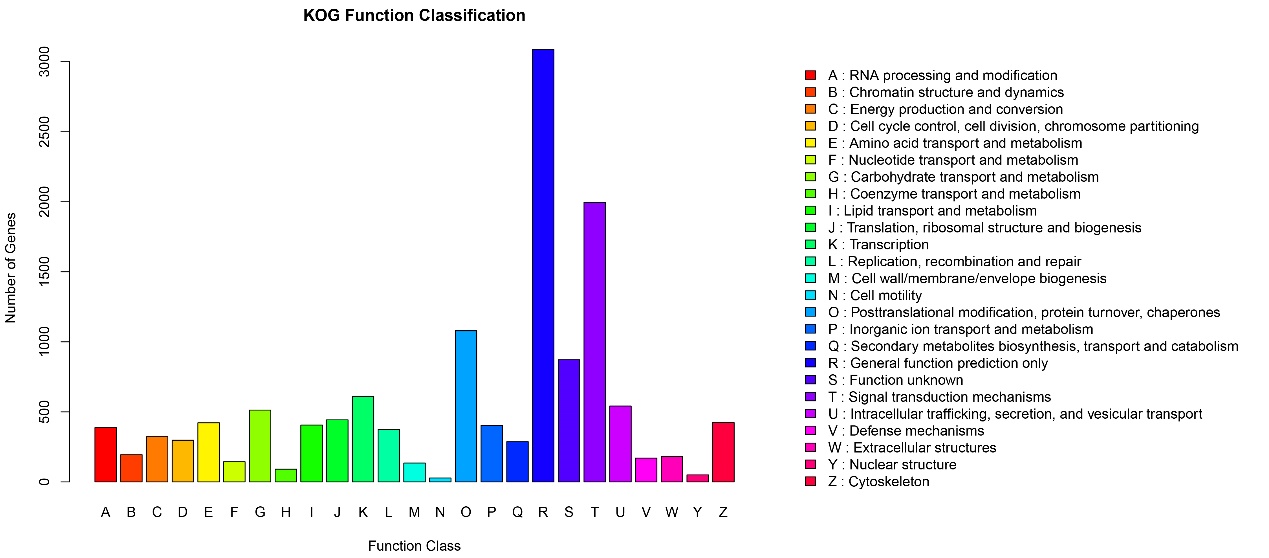
**

**Figure S3**

**
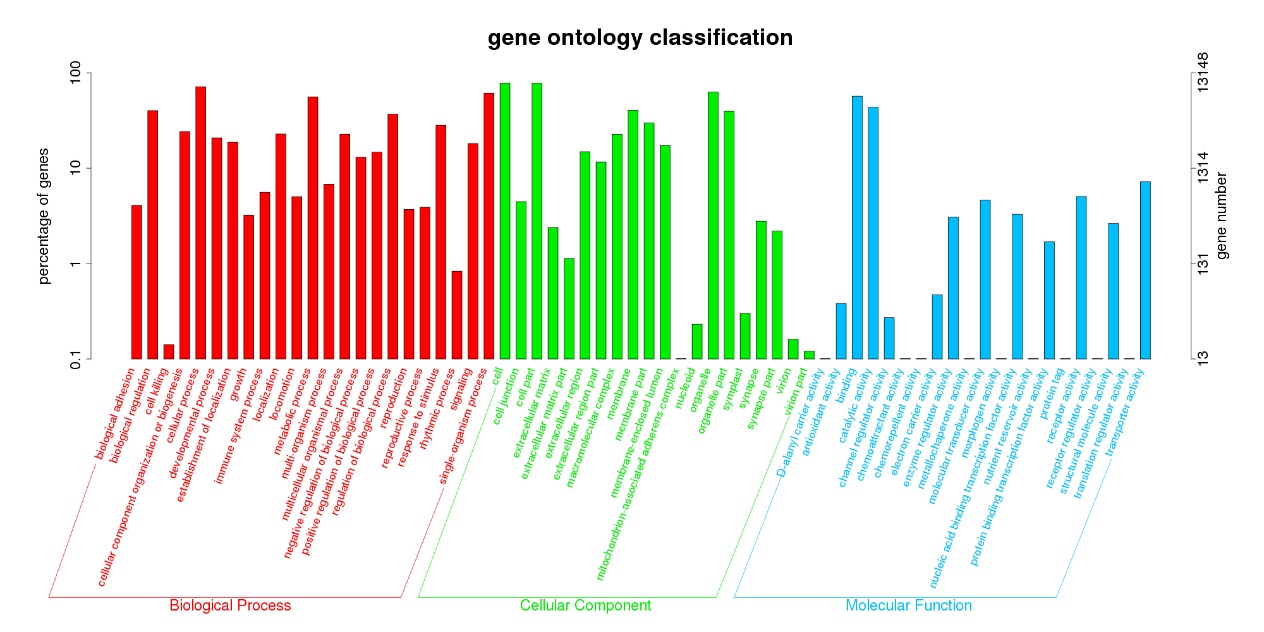
**

**Figure S4**

**
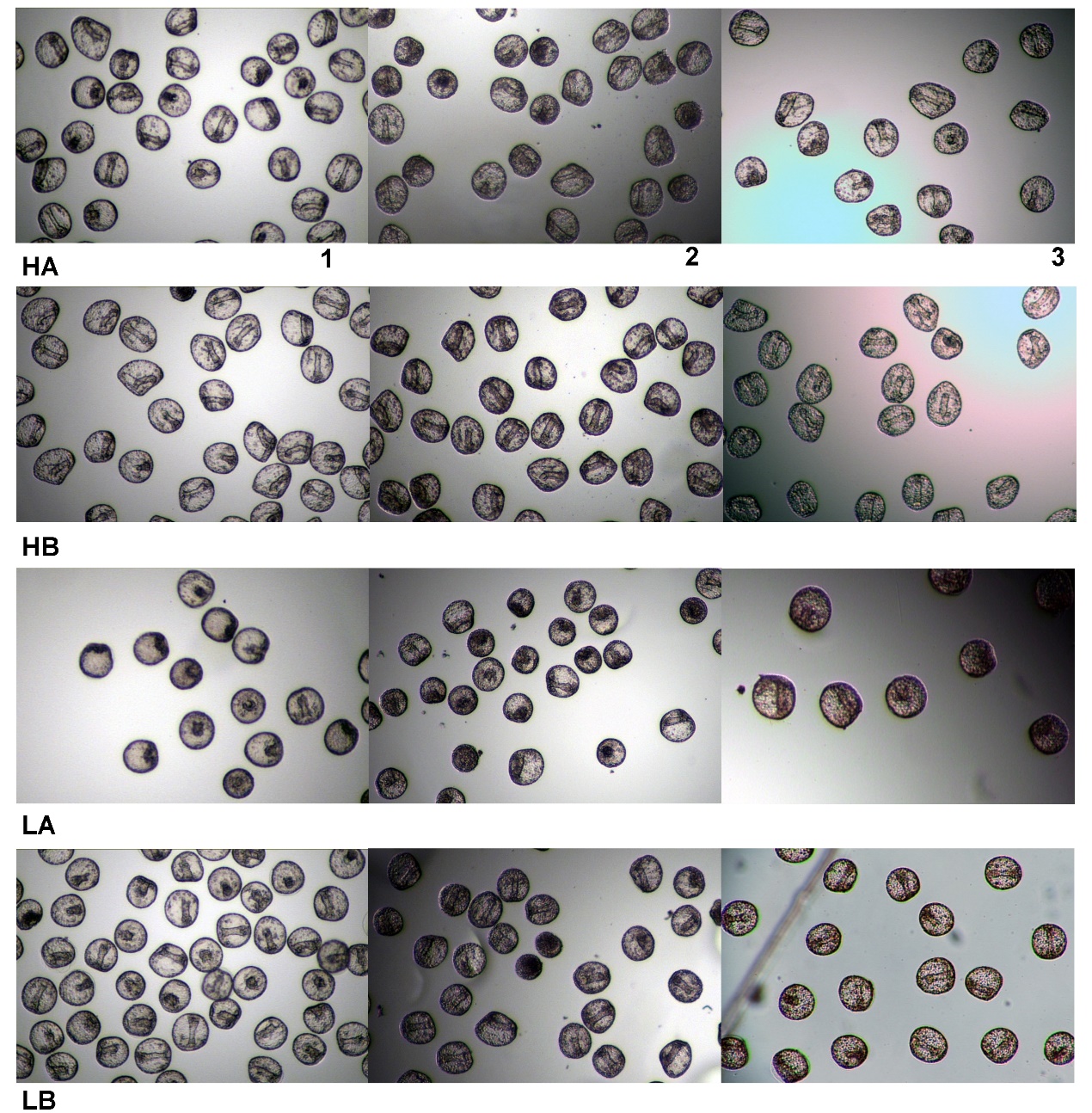
**

**Supplementary Table**

**Table S1** Statistics of sequencing and *de novo* assembling of the transcriptomes of *Strongylocentrotus intermedius* (shown as the average values).

| Sample | Clean reads | Clean bases | Valid ratio | Q30 | GC content |
| --- | --- | --- | --- | --- | --- |
| HA | 42,399,941 | 6,349,561,157 | 94.67% | 88.69% | 39.67% |
| HB | 42,072,720 | 6,300,220,552 | 94.06% | 87.94% | 39.67% |
| LA | 42,032,047 | 6,294,062,803 | 94.22% | 88.16% | 39.00% |
| LB | 42,047,087 | 6,296,394,056 | 94.25% | 88.26% | 39.33% |

**Table S2** Statistics of annotations for assembled unigenes of *Strongylocentrotus intermedius*

| Database | NR | SWISSPROT | KOG | KEGG | GO |
| --- | --- | --- | --- | --- | --- |
| Account | 21,873 | 14,547 | 12,050 | 4577 | 13,148 |
| Percentage | 30.36% | 20.19% | 16.72% | 6.35% | 18.25% |

**Table S3** Partial functional enrichments of Gene ontology (GO) terms in differentially expressed genes between the groups.

| **Group** | **Down/Up Regulated** | **GO ID** | **GO term name** | **Category** | **No. transcript** | **padj** |
| --- | --- | --- | --- | --- | --- | --- |
| **HA vs LA** | Down | GO:0005886 | plasma membrane | Cellular component | 31 | 0.003016 |
|  | Down | GO:0005887 | integral component of plasma membrane | Cellular component | 19 | 6.09E-05 |
|  | Down | GO:0005576 | extracellular region | Cellular component | 15 | 0.001032 |
|  | Down | GO:0005615 | extracellular space | Cellular component | 13 | 0.002004 |
|  | Down | GO:0030054 | cell junction | Cellular component | 9 | 0.002425 |
|  | Down | GO:0005509 | calcium ion binding | Molecular function | 25 | 7.79E-08 |
|  | Down | GO:0004252 | serine-type endopeptidase activity | Molecular function | 5 | 0.003152 |
|  | Up | GO:0005730 | nucleolus | Cellular component | 3 | 0.002639 |
|  | Up | GO:0003677 | DNA binding | Molecular function | 3 | 0.014785 |
| **HB vs LB** | Down | GO:0005886 | plasma membrane | Cellular component | 44 | 0.003137 |
|  | Down | GO:0005576 | extracellular region | Cellular component | 23 | 5.47E-05 |
|  | Down | GO:0005615 | extracellular space | Cellular component | 18 | 0.001613 |
|  | Down | GO:0045202 | synapse | Cellular component | 8 | 0.001024 |
|  | Down | GO:0043005 | neuron projection | Cellular component | 7 | 0.005647 |
|  | Down | GO:0005509 | calcium ion binding | Molecular function | 32 | 8.90E-08 |
|  | Down | GO:0004252 | serine-type endopeptidase activity | Molecular function | 8 | 0.00045 |
|  | Down | GO:0008237 | metallopeptidase activity | Molecular function | 7 | 0.000113 |
|  | Down | GO:0007155 | cell adhesion | Biological process | 9 | 0.019045 |
|  | Down | GO:0006508 | proteolysis | Biological process | 7 | 0.003152 |
|  | Down | GO:0007186 | G-protein coupled receptor signaling pathway | Biological process | 7 | 0.003152 |
|  | Up | GO:0005634 | nucleus | Cellular component | 17 | 0.000281 |
|  | Up | GO:0005730 | nucleolus | Cellular component | 9 | 1.48E-05 |
|  | Up | GO:0044822 | poly(A) RNA binding | Molecular function | 7 | 0.000312 |
|  | Up | GO:0003677 | DNA binding | Molecular function | 7 | 0.004152 |
|  | Up | GO:0006351 | transcription, DNA-templated | Biological process | 6 | 0.021678 |
|  | Down | GO:0005886 | plasma membrane | Cellular component | 60 | 0.001487 |
| **HB vs HA** | Down | GO:0043005 | neuron projection | Cellular component | 12 | 0.0001 |
|  | Down | GO:0005509 | calcium ion binding | Molecular function | 43 | 1.11E-09 |
|  | Down | GO:0004252 | serine-type endopeptidase activity | Molecular function | 9 | 0.000912 |
|  | Down | GO:0004930 | G-protein coupled receptor activity | Molecular function | 7 | 0.028091 |
|  | Down | GO:0005328 | neurotransmitter: sodium symporter activity | Molecular function | 6 | 6.96E-05 |
|  | Down | GO:0007268 | synaptic transmission | Biological process | 10 | 9.60E-05 |
|  | Down | GO:0006814 | sodium ion transport | Biological process | 7 | 0.002526 |
|  | Down | GO:0034220 | ion transmembrane transport | Biological process | 6 | 0.013707 |
|  | Down | GO:0050896 | response to stimulus | Biological process | 6 | 0.000692 |
|  | Down | GO:0035725 | sodium ion transmembrane transport | Biological process | 6 | 0.000912 |
|  | Down | GO:0007399 | nervous system development | Biological process | 6 | 0.048978 |
|  | Up | GO:0005634 | nucleus | Cellular component | 45 | 0.008889 |
|  | Up | GO:0003964 | RNA-directed DNA polymerase activity | Molecular function | 35 | 2.63E-11 |
|  | Up | GO:0003677 | DNA binding | Molecular function | 28 | 7.41E-06 |
|  | Up | GO:0008270 | zinc ion binding | Molecular function | 22 | 0.000884 |
|  | Up | GO:0004190 | aspartic-type endopeptidase activity | Molecular function | 16 | 2.26E-09 |
|  | Up | GO:0004519 | endonuclease activity | Molecular function | 13 | 3.37E-06 |
|  | Up | GO:0003676 | nucleic acid binding | Molecular function | 13 | 3.23E-05 |
|  | Up | GO:0003723 | RNA binding | Molecular function | 9 | 0.023312 |
|  | Up | GO:0003887 | DNA-directed DNA polymerase activity | Molecular function | 8 | 9.01E-05 |
|  | Up | GO:0015074 | DNA integration | Biological process | 18 | 2.31E-09 |
|  | Up | GO:0006278 | RNA-dependent DNA replication | Biological process | 16 | 3.49E-06 |
|  | Up | GO:0006313 | transposition, DNA-mediated | Biological process | 13 | 0.00019 |
|  | Down | GO:0005886 | plasma membrane | Cellular component | 41 | 0.006991 |
| **LB vs LA** | Down | GO:0005509 | calcium ion binding | Molecular function | 29 | 2.77E-06 |
|  | Down | GO:0043565 | sequence-specific DNA binding | Molecular function | 8 | 0.026187 |
|  | Down | GO:0004252 | serine-type endopeptidase activity | Molecular function | 5 | 0.01312 |
|  | Down | GO:0015293 | symporter activity | Molecular function | 5 | 0.019203 |
|  | Down | GO:0007601 | visual perception | Biological process | 6 | 0.001465 |
|  | Down | GO:0045893 | positive regulation of transcription, DNA-templated | Biological process | 6 | 0.04555 |
|  | Down | GO:0035725 | sodium ion transmembrane transport | Biological process | 5 | 0.001493 |
|  | Down | GO:0007399 | nervous system development | Biological process | 5 | 0.031515 |
|  | Up | GO:0005634 | nucleus | Cellular component | 38 | 0.007743 |
|  | Up | GO:0003964 | RNA-directed DNA polymerase activity | Molecular function | 24 | 2.64E-06 |
|  | Up | GO:0003677 | DNA binding | Molecular function | 19 | 0.001804 |
|  | Up | GO:0008270 | zinc ion binding | Molecular function | 15 | 0.014151 |
|  | Up | GO:0004190 | aspartic-type endopeptidase activity | Molecular function | 11 | 7.84E-06 |
|  | Up | GO:0004519 | endonuclease activity | Molecular function | 10 | 0.00011 |
|  | Up | GO:0003676 | nucleic acid binding | Molecular function | 10 | 0.000578 |
|  | Up | GO:0005525 | GTP binding | Molecular function | 7 | 0.00631 |
|  | Up | GO:0003723 | RNA binding | Molecular function | 7 | 0.037952 |
|  | Up | GO:0007275 | multicellular organismal development | Biological process | 6 | 0.028813 |
|  | Up | GO:0015074 | DNA integration | Biological process | 13 | 3.14E-06 |
|  | Up | GO:0006313 | transposition, DNA-mediated | Biological process | 7 | 0.017814 |
|  | Up | GO:0006278 | RNA-dependent DNA replication | Biological process | 7 | 0.021486 |
